# EQ-5D population norms for England: comparing the UK and England EQ-5D-5L value sets, the former NICE reference-case mapping and the crosswalk

**DOI:** 10.1007/s11136-026-04417-3

**Published:** 2026-11-06

**Authors:** Ling Jie Cheng, Xun Li, Annushiah Vasan Thakumar

**Affiliations:** 1 National Perinatal Epidemiology Unit, Nuffield Department of Women’s & Reproductive Health, University of Oxford, Oxford, United Kingdom; 2 Alice Lee Centre for Nursing Studies, Yong Loo Lin School of Medicine, National University of Singapore, Singapore, Singapore; 3 School of Engineering, Computing, and Mathematics, Oxford Brookes University, Oxford, United Kingdom; 4 School of Pharmacy, Faculty of Health & Medical Sciences, Taylor’s University, Selangor, Malaysia

**Keywords:** EQ-5D-5L, Population norms, Value set, Health Survey for England, Health-related quality of life, Health inequalities

## Abstract

**Purpose:**

To generate EQ-5D-5L population norms for adults in England using the new UK value set, and to compare, within respondents, the UK and England EQ-5D-5L value sets, the former National Institute for Health and Care Excellence (NICE) reference-case mapping, and the crosswalk.

**Methods:**

We analysed the Health Survey for England 2014 (EQ-5D-3L; *n* = 7,085) and 2017 (EQ-5D-5L; *n* = 7,169). EQ-5D-5L responses were scored with both value sets and converted to the UK EQ-5D-3L value set using the mapping and the crosswalk. Agreement was assessed against a prespecified equivalence margin of 0.030, and differences decomposed by dimension.

**Results:**

Mean index scores were 0.867 (SD 0.223) under the UK EQ-5D-3L value set, and 0.891 (SD 0.206) and 0.885 (SD 0.187) under the UK and England EQ-5D-5L value sets. The latter two were equivalent at the population level (mean difference 0.006, 90% CI 0.005 to 0.007), though the mean absolute difference was 0.020. They diverged for severe states: among respondents reporting very bad self-rated health, means were 0.243 and 0.313, with the severe-state divergence attributable mainly to pain. The deprivation gradient was larger under the UK value set (0.068 versus 0.059; difference 0.009, 95% CI 0.006 to 0.012), though standardised gradients were near-identical (0.33 and 0.32).

**Conclusion:**

The two EQ-5D-5L value sets produced equivalent norms at the population level but diverged for severe health states, with the larger absolute gradient under the UK value set largely reflecting its wider dispersion. With the UK value set used for NICE reference-case analyses from 27 August 2026, these norms provide reference values on the scale now used in NICE evaluations.

**Supplementary Information:**

The online version contains supplementary material available at https://doi.org/10.1007/s11136-026-04417-3.

## Abstract (plain language summary)

The EQ-5D is a short questionnaire that measures health across five areas: mobility, self-care, usual activities, pain or discomfort, and anxiety or depression. Responses are converted into a single score using a ‘value set’ that reflects how the general public views different health problems. The United Kingdom has a new, officially recommended value set for the five-level version of the EQ-5D, but no reference scores for the general population in England have been published using it. Such reference scores, called population norms, help researchers and decision-makers judge how healthy a patient group is compared with the general public. Using data from the Health Survey for England, we produced average scores by age, sex, and social and health circumstances, and compared the new UK value set with the earlier England value set in the same people. The two value sets gave very similar average scores overall, but differences for individual respondents were larger than the overall average difference suggested because the value sets did not always differ in the same direction. In particular, the new UK value set gave lower scores for people in very poor health, especially when severe pain or discomfort was reported. It also showed a wider absolute gap between people living in the least and most deprived areas, although the relative size of this socioeconomic gradient was very similar between the two value sets. These findings suggest that the two value sets produce broadly similar population-level norms but are not interchangeable for all health states. This matters when EQ-5D scores are used to assess treatment value, compare patient groups with the general population, or study health inequalities.

**Supplementary Information:**

The online version contains supplementary material available at https://doi.org/10.1007/s11136-026-04417-3.

## Introduction

The EQ-5D instrument is one of the most widely used preference-based measures of health-related quality of life worldwide and underpins the estimation of quality-adjusted life-years in health technology assessment (HTA) [1, 2]. Two versions of the descriptive system are in current use. The original three-level version (EQ-5D-3L) classifies health into 243 states across five dimensions: mobility, self-care, usual activities, pain/discomfort, and anxiety/depression, each with three severity levels. The five-level version (EQ-5D-5L) extends this to 3,125 states and was developed to improve sensitivity and reduce ceiling effects [3]. Subsequent evidence suggests that the EQ-5D-5L offers improved discriminatory power and a less pronounced ceiling effect than the EQ-5D-3L across general and patient populations [4, 5]. Reference values describing the distribution of EQ-5D index and EQ VAS scores in the general population, commonly termed population norms, are essential complements to these instruments. They allow analysts to benchmark patient-reported outcomes against values for the general population, populate background utility in decision-analytic models, and quantify the population burden of disease [6, 7].

In the United Kingdom, the EQ-5D has been included in national health surveys for more than three decades and has supported successive sets of population reference data [8, 9]. For most of this period, England-specific norms have been generated from the EQ-5D-3L, scored using the UK EQ-5D-3L value set, which was elicited by time trade-off (TTO) [10]. The Health Survey for England (HSE), an annual nationally representative survey of people living in private households in England [11], included the EQ-5D-3L until 2014 and switched to the EQ-5D-5L from 2017 onwards. For the EQ-5D-5L, an England value set was published in 2018 using composite TTO and discrete choice experiment data from a representative English sample [12, 13]. A subsequent independent quality-assurance review commissioned by the Department of Health and Social Care identified concerns about aspects of the underlying data collection and the modelling of the England EQ-5D-5L value set, principally the conduct of some composite TTO interviews and the treatment of logically inconsistent responses. These findings remain contested, and the value set has continued to be used widely in English applied work, but they prompted the search for a decision-maker-preferred alternative [14]. The National Institute for Health and Care Excellence (NICE) therefore recommended continued use of the UK EQ-5D-3L value set [15], applied to EQ-5D-5L responses via a published mapping algorithm [16–18], for technology appraisals. For most of the intervening period, England therefore lacked a directly elicited, decision-maker-preferred EQ-5D-5L value set, and existing England-based EQ-5D-5L norms have been reported using the England EQ-5D-5L value set [12] or the EQ-5D-3L mapping [16–18].

The three value sets compared here represent successive phases in health-state valuation. The 1997 UK EQ-5D-3L value set emerged from the Measurement and Valuation of Health study, a landmark large-scale general-population valuation study [10, 19, 20], and was subsequently recommended for NICE reference-case analyses for more than two decades [21]. Its methods later attracted criticism. The time trade-off procedures for states considered worse than dead and better than dead differed [22], raising concerns about comparability around the dead anchor [23]; the preferred model was largely additive and included an additional N3 term [10]; and the valuation and modelling methods predated subsequent methodological developments [24, 25]. Sustained methodological research led to a standardised valuation protocol incorporating composite time trade-off, which underpins both EQ-5D-5L value sets examined here [12, 24–26]. However, the underlying valuation and modelling framework has also been questioned. Recent critiques have asked whether stated-preference valuation supports the interpersonal comparisons and aggregation required to estimate quality-adjusted life-years [27], and whether the modelling assumptions used to extrapolate from a sampled subset of health states to the full descriptive system are adequately justified and empirically validated [14]. Although these concerns arise broadly across the three value sets, their nature and extent are not identical, and they do not, by themselves, favour one value set over another. Nevertheless, they affect interpretation: differences between value sets may reflect differences in their descriptive systems, sampling frames, elicitation protocols, modelling choices, and periods of preference elicitation, rather than differences in accuracy against an external gold standard.

Against this background, a new UK EQ-5D-5L value set has been published [26, 28]. It was derived using composite time trade-off data from a representative sample of 1,200 adults across England, Wales, Scotland, and Northern Ireland and modelled using a random-effects Tobit specification [26, 28]. NICE has since adopted the new value set. From 27 August 2026, the EQ-5D-5L descriptive system and UK EQ-5D-5L value set are to be used for all adult reference-case analyses under an interim methods statement that replaces the 2019 position statement and takes precedence over the existing methods manuals [29]. Compared with the UK EQ-5D-3L value set, the UK EQ-5D-5L value set gives greater relative weight to anxiety/depression and usual activities, while the value assigned to the worst health state is broadly comparable [26]. Because the new value set reflects UK rather than England-only preferences and uses elicitation and modelling approaches that differ from those used for the England EQ-5D-5L value set [12], it may alter the distribution of index scores, population reference values, age- and sex-specific profiles, and socioeconomic gradients in ways that matter for health technology assessment and population health analysis. To our knowledge, no EQ-5D-5L population norms for community-dwelling adults in England have been published using the new UK value set, and no study has directly compared norms generated using the UK and England EQ-5D-5L value sets, the former NICE reference-case mapping, and the crosswalk within a nationally representative sample.

Using two waves of the HSE (HSE 2014 for the EQ-5D-3L and HSE 2017 for the EQ-5D-5L), we therefore aimed to: (i) update population norms for community-dwelling adults in England for the EQ-5D index and EQ VAS by age, sex, and key sociodemographic and health subgroups; (ii) generate the first set of EQ-5D-5L norms for England using the UK EQ-5D-5L value set; (iii) test, within the same respondents, whether these are equivalent to norms generated using the England EQ-5D-5L value set against a prespecified margin of 0.030, and identify the dimensions responsible for any divergence; and (iv) test whether the socioeconomic gradient in EQ-5D index scores differs between the two EQ-5D-5L value sets, and whether any difference persists once their differing dispersion is accounted for. Aims (iii) and (iv) are within-respondent comparisons, since both EQ-5D-5L value sets score the same responses from the same individuals, so cohort and descriptive system are held constant by design.

## Methods

### Study design and data sources

We conducted a secondary analysis of two waves of the Health Survey for England (HSE), an annual cross-sectional survey. Each annual HSE provides nationally representative estimates of health and health-related behaviours among people living in private households in England. The survey uses a multistage stratified probability sample of addresses drawn from the Postcode Address File. Adults aged 16 years and older in selected households are invited to a face-to-face interviewer-administered visit, and consenting respondents are subsequently visited by a research nurse for clinical measurements. The EQ-5D is not part of the interviewer-administered module: in both waves it was completed by the respondent in the adult self-completion booklet. Mode of administration is therefore constant across the two waves. Individuals living in communal establishments are excluded [11, 30]. Around 8,000 adults are interviewed in a typical year; the achieved adult sample was 8,077 in HSE 2014 and 7,997 in HSE 2017 [31, 32].

We used the 2014 wave, which included the three-level version of the EQ-5D (EQ-5D-3L), and the 2017 wave, which included the five-level version (EQ-5D-5L). The analytic sample in each wave comprised adults aged 16 years and over with a complete response on all five EQ-5D dimensions and a positive core interview weight [33, 34]. This yielded 7,085 respondents in HSE 2014 (EQ-5D-3L) and 7,169 in HSE 2017 (EQ-5D-5L). The derivation, with counts at every step, is reported in Supplementary Table S1. The EQ VAS, in which respondents rate their health today on a vertical scale from 0 (worst imaginable health) to 100 (best imaginable health) [35], was available for 6,490 respondents in HSE 2014 and 7,037 respondents in HSE 2017. The HSE data are publicly available and fully anonymised; therefore, this analysis did not require additional ethical approval.

### Variables

Sociodemographic and health variables were extracted from each wave using harmonised definitions. These included age in 10-year groups (16–24, 25–34, 35–44, 45–54, 55–64, 65–74, and ≥ 75 years), sex, ethnicity (White versus non-White, owing to small numbers in some categories), marital status (married/cohabiting; single, never married; separated/divorced/widowed), highest educational qualification (degree or higher versus below degree), employment status (employed; retired; unemployed; other economically inactive, including students, those looking after the home or family, those permanently unable to work, and other groups), Government Office Region, and quintile of the Index of Multiple Deprivation (IMD; Q1 = least deprived, Q5 = most deprived). The IMD 2010 was used in HSE 2014 and the IMD 2015 in HSE 2017, consistent with the IMD vintage available at the time of each survey. Self-reported general health was recorded on the standard five-point scale: very good, good, fair, bad, and very bad. Longstanding illness and limiting longstanding illness were defined according to the standard HSE questions.

### EQ-5D scoring and value sets

EQ-5D-3L responses from HSE 2014 were scored using the UK EQ-5D-3L value set, elicited by time trade-off (TTO) [10]. The resulting index ranges from −0.594 to 1.000.

EQ-5D-5L responses from HSE 2017 were scored using two value sets to enable comparison. First, we used the UK EQ-5D-5L value set [26], derived using composite TTO with a representative sample of 1,200 UK adults across the four UK nations and modelled using a random-effects Tobit specification. This value set is recommended by the National Institute for Health and Care Excellence (NICE) for adult reference-case analyses [36]. The resulting index ranges from −0.567 to 1.000. Second, we used the England EQ-5D-5L value set [12], derived using composite TTO and discrete choice experiment data from a representative English sample of 996 respondents. This remains the most widely cited EQ-5D-5L value set for England. The resulting index ranges from −0.285 to 1.000. The three value sets therefore differ on four axes: the population whose preferences were elicited (UK-wide versus England only), the elicitation task (TTO alone for the EQ-5D-3L value set; composite TTO for the UK EQ-5D-5L value set; composite TTO combined with a discrete choice experiment for the England EQ-5D-5L value set), the modelling approach (a main-effects additive model with an N3 term; a random-effects Tobit; and a hybrid model combining the two elicitation modes), and the resulting scale, whose lower bound ranges from −0.594 to −0.285. Supplementary Table S2 reports the coefficients of all three, together with the provenance of the two conversion routes.

### Statistical analysis

All analyses incorporated the HSE complex survey design, including primary sampling units, strata, and the published core adult interview weight. The weights account for unequal selection probabilities and differential non-response and calibrate the achieved sample to population totals by age, sex, and region [31, 32]. We retained them for overall and subgroup estimates. We also specified primary sampling units and strata to account for clustering and obtain appropriate standard errors and confidence intervals. Estimates were obtained using Stata’s svy prefix (Stata/MP 18.0; StataCorp, College Station, TX, USA). Strata containing a single primary sampling unit were treated as certainty units.

We computed weighted descriptive statistics for the EQ-5D index, including mean, standard deviation, median, and minimum values; the EQ VAS, including mean and standard deviation; and the ceiling effect, defined as the proportion reporting profile 11111, which is the exact complement of the proportion reporting any problem. Means and 95% confidence intervals (CIs) for the EQ-5D index and EQ VAS were generated overall and by age group, sex, age-by-sex stratum, ethnicity, marital status, education, employment, IMD quintile, region, longstanding illness, limiting longstanding illness, and self-rated general health. Weighted dimension-level response frequencies were computed for each instrument, and the 20 most frequent health-state profiles were tabulated.

To examine the independent associations between sociodemographic and health characteristics and EQ-5D outcomes, we fitted survey-weighted ordinary least squares regressions with linearised standard errors. Separate models were estimated for each EQ-5D index outcome: the EQ-5D-3L scored using the UK EQ-5D-3L value set; the EQ-5D-5L scored using the UK and England EQ-5D-5L value sets; and the EQ-5D-5L responses converted to the UK EQ-5D-3L scale using the former NICE mapping and the crosswalk. Separate models were also estimated for the EQ VAS in each survey wave. All regressions adjusted for sex, age group, ethnicity, marital status, education, IMD quintile, employment status, and limiting longstanding illness. Associations were also expressed relative to the standard deviation of each outcome, which permits comparison across dependent variables with different ranges and variances.

To assess agreement between the two EQ-5D-5L value sets, we calculated within-respondent differences in index scores for HSE 2017, defined as the score under the UK EQ-5D-5L value set minus the score under the England EQ-5D-5L value set. We summarised both the signed mean difference and the mean absolute difference, because the signed mean can mask disagreements in opposite directions. These differences were summarised overall and by subgroup. We also visualised the distributions of EQ-5D-5L index scores generated by each value set.

We assessed population-level equivalence using two one-sided tests with a prespecified margin of ± 0.030. We concluded equivalence when the entire 90% CI for the mean within-respondent difference lay within this margin. To identify the sources of disagreement, we decomposed each within-respondent difference into dimension-specific contributions, calculated as the difference between the decrements assigned by the two value sets for each reported dimension level, and summarised these contributions overall and by severity.

### Sensitivity analyses

The EQ-5D index distribution is bounded above at 1 and exhibits a substantial ceiling, which can bias ordinary least squares coefficients. We therefore re-estimated the index regressions using survey-weighted upper-censored Tobit models (svy: tobit), censoring at 1. For the EQ VAS, a scale bounded between 0 and 100, we re-estimated the regressions using a survey-weighted generalised linear model with a binomial family scaled to 100 and a logit link (svy: glm). This fractional-response specification respects the bounded range of the scale. These analyses are reported as sensitivity analyses; primary inferences are drawn from the survey-weighted ordinary least squares models.

## Results

### Sample characteristics

Sociodemographic and health characteristics of the two analytic samples are summarised in Table 1. Weighted characteristics were similar across the two waves. Women comprised 51.5% of HSE 2014 and 51.8% of HSE 2017 respondents, and the weighted proportion who were White was 88.3% and 86.4%, respectively. The two waves were closely comparable in weighted age structure, with 20.1% of respondents aged ≥ 65 years in HSE 2014 and 21.7% in HSE 2017. The weighted prevalence of limiting longstanding illness was 21.4% in HSE 2014 and 24.1% in HSE 2017. The proportion with a degree or higher was 27.5% and 30.6%, respectively, while 57.7% and 58.1% were employed. The IMD-quintile distribution was approximately even in both waves, ranging from 18% to 22% across quintiles.

**Table 1:** Sociodemographic and health characteristics of the analytic samples, Health Survey for England 2014 and 2017

Characteristic	HSE 2014 (EQ-5D-3L)			HSE 2017 (EQ-5D-5L)		
	*n*	Unweighted %	Weighted %	*n*	Unweighted %	Weighted %
Analytic sample, n	7,085			7,169		
Age group, years						
16–24	681	9.6	14.2	560	7.8	12.9
25–34	991	14.0	16.9	977	13.6	17.2
35–44	1,251	17.7	16.7	1,196	16.7	15.9
45–54	1,329	18.8	17.9	1,218	17.0	17.6
55–64	1,075	15.2	14.3	1,243	17.3	14.8
65–74	1,064	15.0	11.8	1,124	15.7	12.5
75+	694	9.8	8.3	851	11.9	9.2
Missing	0	0	—	0	0	—
Sex						
Male	3,111	43.9	48.5	3,121	43.5	48.2
Female	3,974	56.1	51.5	4,048	56.5	51.8
Missing	0	0	—	0	0	—
Ethnicity						
White	6,369	89.9	88.3	6,340	88.4	86.4
Non-White	710	10.0	11.7	818	11.4	13.6
Missing	6	0.1	—	11	0.2	—
Marital status						
Married or cohabiting	4,526	63.9	62.2	4,700	65.6	63.9
Single	1,361	19.2	23.6	1,308	18.2	22.7
Separated, divorced or widowed	1,196	16.9	14.2	1,161	16.2	13.3
Missing	2	0.0	—	0	0	—
Education						
Degree or higher	1,862	26.3	27.5	2,098	29.3	30.6
Below degree	5,213	73.6	72.5	5,058	70.6	69.4
Missing	10	0.1	—	13	0.2	—
Employment status						
Employed	3,907	55.1	57.7	3,892	54.3	58.1
Retired	1,830	25.8	21.3	2,006	28.0	22.0
Unemployed	275	3.9	4.7	115	1.6	1.8
Other economically inactive	1,068	15.1	16.3	1,155	16.1	18.1
Missing	5	0.1	—	1	0.0	—
IMD deprivation quintile						
Q1 (least deprived)	1,603	22.6	22.3	1,500	20.9	20.1
Q2	1,427	20.1	19.6	1,552	21.6	21.5
Q3	1,388	19.6	19.5	1,372	19.1	20.0
Q4	1,383	19.5	20.2	1,406	19.6	19.9
Q5 (most deprived)	1,284	18.1	18.4	1,339	18.7	18.5
Missing	0	0	—	0	0	—
Longstanding illness						
Yes	2,920	41.2	38.4	3,296	46.0	42.7
No	4,160	58.7	61.6	3,869	54.0	57.3
Missing	5	0.1	—	4	0.1	—
Limiting longstanding illness						
Yes	1,663	23.5	21.4	1,895	26.4	24.1
No	5,417	76.5	78.6	5,270	73.5	75.9
Missing	5	0.1	—	4	0.1	—
Self-rated general health						
Very good	2,339	33.0	34.2	2,353	32.8	34.5
Good	3,026	42.7	43.2	2,975	41.5	42.0
Fair	1,229	17.3	16.3	1,272	17.7	16.5
Bad	371	5.2	4.8	408	5.7	5.1
Very bad	118	1.7	1.5	159	2.2	1.9
Missing	2	0.0	—	2	0.0	—
EQ VAS						
n with a valid response	6,490			7,037		
Missing	595			132		

Estimates are survey-weighted using the core interview weight and the complex survey design

Weighted percentages are estimated among respondents with a non-missing value for that variable, so category counts and the missing count sum to the analytic sample in every block

*HSE* Health Survey for England, *IMD* Index of Multiple Deprivation

### Overall EQ-5D index scores and EQ VAS

Survey-weighted estimates of the EQ-5D index and EQ VAS are presented in Table 2. Under the UK EQ-5D-3L value set, the mean EQ-5D-3L index in HSE 2014 was 0.867 (SD 0.223; 95% CI 0.860 to 0.873). Overall, 59.6% of respondents reported full health, corresponding to profile 11111, and 1.5% scored below zero. The mean EQ VAS was 78.5 (SD 17.9).

**Table 2:** EQ-5D index scores and EQ VAS by descriptive system and value set, Health Survey for England 2014 and 2017

Outcome	HSE 2014	HSE 2017			
	UK EQ-5D-3L	UK EQ-5D-5L	England EQ-5D-5L	UK EQ-5D-3L (mapped)	UK EQ-5D-3L (crosswalk)
Analytic sample, n	7,085	7,169	7,169	7,169	7,169
Mean (SD)	0.867 (0.223)	0.891 (0.206)	0.885 (0.187)	0.833 (0.222)	0.838 (0.218)
95% CI for the mean	0.860 to 0.873	0.885 to 0.896	0.880 to 0.890	0.827 to 0.839	0.832 to 0.844
Full health, profile 11111, %	59.6	44.6	—	—	—
Index value below zero, %	1.5	1.2	0.7	1.4	1.3
Minimum observed value	−0.594	−0.523	−0.246	−0.445	−0.511
EQ VAS, mean (SD)	78.5 (17.9)	79.2 (18.7)	—	—	—
EQ VAS, n with a valid response	6,490	7,037	—	—	—

Estimates are survey-weighted. Index values are reported to three decimal places

Full health is defined by the response profile 11111 and is a property of the descriptive system rather than of the value set; it is therefore reported once per wave. The complementary any-problem proportion is not reported, being its exact complement. The EQ VAS is reported once per wave for the same reason

The two right-hand columns score the same EQ-5D-5L responses on the UK EQ-5D-3L value set. Scores on the UK EQ-5D-3L scale, including both conversion routes, are not directly comparable in level with scores on an EQ-5D-5L scale

Value sets: UK EQ-5D-3L, Dolan 1997; UK EQ-5D-5L, Rowen et al. 2026; England EQ-5D-5L, Devlin et al. 2018. Where EQ-5D-5L responses are converted to the UK EQ-5D-3L value set, mapped denotes the NICE Decision Support Unit algorithm and crosswalk denotes the algorithm of van Hout et al. (2012)

The mapped column is a conditional expectation given the response profile, age and sex; its maximum is 0.9885, so it has no ceiling.

*HSE* Health Survey for England, *SD* Standard deviation, *CI* Confidence interval

In HSE 2017, the mean EQ-5D-5L index was 0.891 (SD 0.206; 95% CI 0.885 to 0.896) under the UK EQ-5D-5L value set and 0.885 (SD 0.187; 95% CI 0.880 to 0.890) under the England EQ-5D-5L value set, an overall within-respondent difference of 0.006 (95% CI 0.005 to 0.007). Converting the same responses to the UK EQ-5D-3L value set gave 0.833 (SD 0.222) under the former NICE reference-case mapping and 0.838 (SD 0.218) under the crosswalk [37]. Those two routes sit on a different scale from the EQ-5D-5L columns and are not directly comparable with them. All four scoring routes, five of the six pairwise contrasts, and the crosswalk-mapping contrast by severity are reported in Table 3.

**Table 3:** EQ-5D-5L responses scored four ways: two directly elicited value sets, the reference-case mapping and the crosswalk, Health Survey for England 2017

Scoring route	Scale	Mean	95% CI
Panel A. Population mean under each scoring route			
UK EQ-5D-5L	EQ-5D-5L	0.891	0.885 to 0.896
England EQ-5D-5L	EQ-5D-5L	0.885	0.880 to 0.890
UK EQ-5D-3L (mapped)	EQ-5D-3L	0.833	0.827 to 0.839
UK EQ-5D-3L (crosswalk)	EQ-5D-3L	0.838	0.832 to 0.844
Panel B. Pairwise differences			
Contrast	Comparison	Difference	95% CI
Mapped minus England EQ-5D-5L	Cross-scale	−0.0518	−0.0532 to −0.0505
Mapped minus UK EQ-5D-5L	Cross-scale	−0.0576	−0.0592 to −0.0561
Crosswalk minus England EQ-5D-5L	Cross-scale	−0.0471	−0.0486 to −0.0455
Crosswalk minus UK EQ-5D-5L	Cross-scale	−0.0529	−0.0547 to −0.0512
Crosswalk minus mapped	Like for like	0.0047	0.0035 to 0.0060
Panel C. Crosswalk minus mapped, by number of dimensions at level 4 or worse			
Severe dimensions	n	Difference	95% CI
0	6,439	0.0036	0.0029 to 0.0043
1	322	−0.0127	−0.0340 to 0.0086
2	182	0.0088	−0.0105 to 0.0282
3	132	0.0714	0.0548 to 0.0880
4	70	0.0638	0.0450 to 0.0825
5	24	‡	‡

All four scoring routes score the same EQ-5D-5L responses from the same 7,169 respondents, so every contrast is within respondent and is attributable entirely to the scoring route

Cross-scale contrasts compare a score on the UK EQ-5D-3L scale with one on an EQ-5D-5L scale. They describe what would be obtained under one scoring convention rather than another and are not evidence that either value set is more accurate

The crosswalk versus mapped contrast is the only like-for-like comparison, as both routes use the UK EQ-5D-3L scale. Against an equivalence margin of ±0.030, equivalence is established at the population level. In Panel C, the difference exceeds the margin for three and four dimensions rated level 4 or worse, whereas equivalence is not established for one such dimension

Value sets: UK EQ-5D-3L, Dolan 1997; UK EQ-5D-5L, Rowen et al. 2026; England EQ-5D-5L, Devlin et al. 2018. Where EQ-5D-5L responses are converted to the UK EQ-5D-3L value set, mapped denotes the NICE Decision Support Unit algorithm and crosswalk denotes the algorithm of van Hout et al. (2012)

The mapped column is a conditional expectation given the response profile, age and sex; its maximum is 0.9885, so it has no ceiling. Its standard deviation, minimum and proportion below zero are reported in Table 2. Scores on the UK EQ-5D-3L scale are not directly comparable in level with scores on an EQ-5D-5L scale

*CI* Confidence interval

‡ suppressed, fewer than 25 unweighted observations.

The ceiling effect was lower in the HSE 2017 EQ-5D-5L sample than in the HSE 2014 EQ-5D-3L sample: 44.6% versus 59.6%. The mean EQ VAS in HSE 2017 was 79.2 (SD 18.7). The minimum observed index value was −0.594 under the UK EQ-5D-3L value set, −0.523 under the UK EQ-5D-5L value set, −0.246 under the England EQ-5D-5L value set, −0.445 under the former NICE reference-case mapping and −0.511 under the crosswalk.

### Index scores by age and sex

**Fig. 1:**
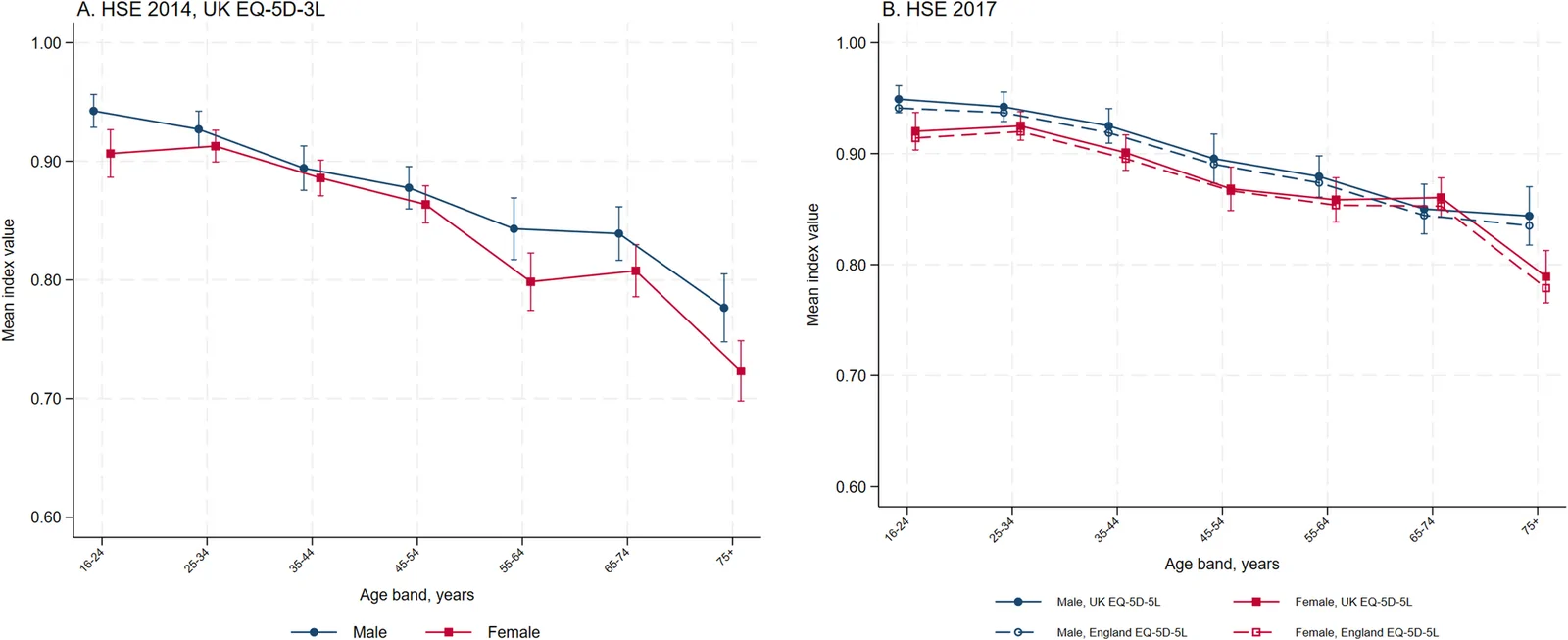
Survey-weighted mean EQ-5D index scores by age group and sex, with 95% confidence intervals. **A** Health Survey for England 2014 (n = 7,085), EQ-5D-3L scored with the UK EQ-5D-3L value set. **B** Health Survey for England 2017 (n = 7,169), EQ-5D-5L scored with the UK EQ-5D-5L value set (solid lines) and the England EQ-5D-5L value set (dashed lines). The same estimates are tabulated in Table 4. Both panels use the standard Health Survey for England ten-year age banding, so the age axis is common; the panels nonetheless differ in cohort, descriptive system and value set, and levels are not comparable between them. Points are offset horizontally within each age band so that overlapping intervals remain legible. Cells containing fewer than 25 unweighted observations are not plotted. Value sets: UK EQ-5D-3L, Dolan 1997; UK EQ-5D-5L, Rowen et al. 2026; England EQ-5D-5L, Devlin et al. 2018

Table 4 and Fig. 1 present survey-weighted mean index scores by age group and sex. Under the UK EQ-5D-3L value set, mean index scores declined from 0.925 among respondents aged 16–24 years to 0.747 among those aged ≥ 75 years. Women had lower mean scores than men in every age stratum, with the largest gap in the oldest group: 0.723 among women versus 0.776 among men.

**Table 4:** Survey-weighted mean EQ-5D index scores by age group and sex, Health Survey for England 2014 and 2017

		HSE 2014		HSE 2017				
Age group, years	Sex	*n*	UK EQ-5D-3L	*n*	UK EQ-5D-5L	England EQ-5D-5L	UK EQ-5D-3L (mapped)	UK EQ-5D-3L (crosswalk)
16–24	Male	317	0.942 (0.103)	248	0.949 (0.068)	0.941 (0.071)	0.909 (0.091)	0.908 (0.103)
16–24	Female	364	0.907 (0.149)	312	0.920 (0.119)	0.914 (0.109)	0.867 (0.139)	0.874 (0.140)
16–24	All	681	0.925 (0.127)	560	0.934 (0.096)	0.927 (0.091)	0.888 (0.117)	0.891 (0.122)
25–34	Male	385	0.927 (0.121)	415	0.942 (0.102)	0.937 (0.098)	0.904 (0.120)	0.907 (0.127)
25–34	Female	606	0.913 (0.179)	562	0.925 (0.142)	0.920 (0.129)	0.880 (0.155)	0.881 (0.163)
25–34	All	991	0.920 (0.151)	977	0.933 (0.123)	0.928 (0.114)	0.892 (0.138)	0.894 (0.146)
35–44	Male	527	0.894 (0.193)	478	0.925 (0.154)	0.919 (0.141)	0.876 (0.175)	0.877 (0.175)
35–44	Female	724	0.886 (0.226)	718	0.901 (0.220)	0.895 (0.196)	0.846 (0.229)	0.850 (0.226)
35–44	All	1,251	0.890 (0.210)	1,196	0.912 (0.189)	0.907 (0.170)	0.860 (0.204)	0.863 (0.202)
45–54	Male	596	0.878 (0.215)	512	0.895 (0.199)	0.890 (0.178)	0.840 (0.212)	0.842 (0.213)
45–54	Female	733	0.864 (0.224)	706	0.868 (0.265)	0.866 (0.233)	0.808 (0.271)	0.817 (0.264)
45–54	All	1,329	0.871 (0.220)	1,218	0.881 (0.233)	0.878 (0.207)	0.824 (0.243)	0.829 (0.239)
55–64	Male	491	0.843 (0.254)	572	0.879 (0.240)	0.874 (0.218)	0.818 (0.256)	0.824 (0.247)
55–64	Female	584	0.798 (0.305)	671	0.858 (0.269)	0.853 (0.240)	0.784 (0.290)	0.796 (0.277)
55–64	All	1,075	0.820 (0.282)	1,243	0.869 (0.255)	0.863 (0.230)	0.801 (0.274)	0.810 (0.263)
65–74	Male	486	0.839 (0.256)	511	0.850 (0.260)	0.844 (0.236)	0.784 (0.270)	0.789 (0.257)
65–74	Female	578	0.808 (0.302)	613	0.860 (0.249)	0.853 (0.225)	0.787 (0.267)	0.792 (0.252)
65–74	All	1,064	0.823 (0.281)	1,124	0.855 (0.256)	0.849 (0.231)	0.786 (0.269)	0.790 (0.255)
75+	Male	309	0.776 (0.274)	385	0.844 (0.277)	0.835 (0.255)	0.773 (0.299)	0.776 (0.294)
75+	Female	385	0.723 (0.289)	466	0.789 (0.283)	0.779 (0.257)	0.705 (0.287)	0.715 (0.269)
75+	All	694	0.747 (0.284)	851	0.812 (0.283)	0.803 (0.258)	0.734 (0.295)	0.741 (0.282)
Overall	Male	3,111	0.881 (0.200)	3,121	0.903 (0.184)	0.897 (0.169)	0.850 (0.201)	0.853 (0.200)
Overall	Female	3,974	0.853 (0.244)	4,048	0.879 (0.225)	0.874 (0.203)	0.817 (0.240)	0.824 (0.233)
Overall	All	7,085	0.867 (0.223)	7,169	0.891 (0.206)	0.885 (0.187)	0.833 (0.222)	0.838 (0.218)

Cells show the survey-weighted mean and standard deviation

Both waves use the standard Health Survey for England ten-year age banding, so the age rows are comparable across waves. The two waves are nonetheless independent cross-sectional samples scored on different scales and should not be read as change over time; see Supplementary Table S10

Value sets: UK EQ-5D-3L, Dolan 1997; UK EQ-5D-5L, Rowen et al. 2026; England EQ-5D-5L, Devlin et al. 2018. Where EQ-5D-5L responses are converted to the UK EQ-5D-3L value set, mapped denotes the NICE Decision Support Unit algorithm and crosswalk denotes the algorithm of van Hout et al. (2012)

The mapped column returns a conditional expectation given the response profile, age and sex, so no value reaches 1.000 (maximum 0.9885) and the column has no ceiling. Averaging within profile removes some within-profile variance, but the effect on dispersion is small: the standard deviation is 0.222, against 0.218 for the crosswalk and 0.223 for the directly scored EQ-5D-3L. Scores on the UK EQ-5D-3L scale are not comparable in level with scores on an EQ-5D-5L scale

*HSE* Health Survey for England

Under the EQ-5D-5L in HSE 2017, mean scores under the UK and England value sets were 0.934 and 0.927, respectively, among respondents aged 16–24 years, and 0.812 and 0.803, respectively, among those aged ≥ 75 years. The two value sets produced very similar age- and sex-specific profiles, with absolute differences in stratum-specific means of 0.010 or less.

### Comparison of the UK and England EQ-5D-5L value sets

A direct comparison of the two EQ-5D-5L value sets within HSE 2017 is shown in Table 5 and Supplementary Figure S1. The mean within-respondent difference was 0.006 (90% CI 0.005 to 0.007, lying entirely within the prespecified equivalence margin of ±0.030), whereas the mean absolute difference was 0.020 (95% CI 0.019 to 0.021), showing greater individual-level disagreement than the signed mean alone suggests. The signed difference was small for most subgroups. Across subgroups, differences ranged from −0.071 to + 0.011.

**Table 5:** EQ-5D-5L index scores under the UK and England value sets, by subgroup, Health Survey for England 2017

Subgroup	Category	*n*	UK EQ-5D-5L	England EQ-5D-5L	Difference	95% CI
Overall	All respondents	7,169	0.891	0.885	0.0058	0.0049 to 0.0067
Age group	16–24	560	0.934	0.927	0.0070	0.0048 to 0.0092
	25–34	977	0.933	0.928	0.0052	0.0031 to 0.0073
	35–44	1,196	0.912	0.907	0.0058	0.0036 to 0.0080
	45–54	1,218	0.881	0.878	0.0033	0.0009 to 0.0058
	55–64	1,243	0.869	0.863	0.0053	0.0028 to 0.0077
	65–74	1,124	0.855	0.849	0.0068	0.0044 to 0.0091
	75+	851	0.812	0.803	0.0096	0.0068 to 0.0125
Sex	Male	3,121	0.903	0.897	0.0062	0.0049 to 0.0074
	Female	4,048	0.879	0.874	0.0055	0.0043 to 0.0067
IMD deprivation quintile	Q1 (least deprived)	1,500	0.919	0.910	0.0090	0.0074 to 0.0106
	Q2	1,552	0.908	0.901	0.0069	0.0054 to 0.0084
	Q3	1,372	0.901	0.893	0.0074	0.0059 to 0.0088
	Q4	1,406	0.869	0.864	0.0051	0.0029 to 0.0073
	Q5 (most deprived)	1,339	0.852	0.852	0.0002	−0.0026 to 0.0030
Limiting longstanding illness	No	5,270	0.956	0.947	0.0087	0.0080 to 0.0094
	Yes	1,895	0.686	0.689	−0.0032	−0.0062 to −0.0002
Self-rated general health	Very good	2,353	0.968	0.962	0.0062	0.0053 to 0.0071
	Good	2,975	0.935	0.924	0.0110	0.0100 to 0.0120
	Fair	1,272	0.811	0.800	0.0111	0.0085 to 0.0136
	Bad	408	0.501	0.529	−0.0276	−0.0358 to −0.0194
	Very bad	159	0.243	0.313	−0.0705	−0.0853 to −0.0557
Employment status	Employed	3,892	0.939	0.930	0.0085	0.0076 to 0.0094
	Retired	2,006	0.835	0.828	0.0074	0.0055 to 0.0093
	Unemployed	115	0.881	0.874	0.0073	0.0001 to 0.0144
	Other economically inactive	1,155	0.805	0.810	−0.0049	−0.0084 to −0.0014

Estimates are survey-weighted. Difference is the UK value set minus the England value set; a positive value indicates a higher index score under the UK value set

Both columns score the same responses from the same respondents, so every contrast is within respondent and is attributable entirely to the value set

The difference in the deprivation gradient was tested as the Q1 versus Q5 contrast from a survey-weighted regression of the within-respondent difference on IMD quintile: 0.009 (95% CI 0.006 to 0.012, P<0.001). The Q1 to Q5 gap, calculated from unrounded means, is 0.068 under the UK value set and 0.059 under the England value set; per standard deviation of the respective index these are 0.33 and 0.32

Value sets: UK EQ-5D-5L, Rowen et al. 2026; England EQ-5D-5L, Devlin et al. 2018

*IMD* Index of Multiple Deprivation, *CI* Confidence interval

The largest absolute difference was observed among respondents reporting very bad self-rated general health. In this group, the mean score was 0.243 under the UK value set and 0.313 under the England value set, a difference of −0.071 (95% CI −0.085 to −0.056). Among respondents reporting bad self-rated general health, the corresponding means were 0.501 and 0.529, a difference of −0.028 (95% CI −0.036 to −0.019). Among respondents with limiting longstanding illness, the corresponding means were 0.686 and 0.689, a difference of −0.0032 (95% CI  −0.0062 to −0.0002). In the most deprived IMD quintile the two value sets were indistinguishable, at 0.852 under both (difference 0.000, 95% CI −0.003 to 0.003), whereas in the least deprived quintile the UK value set gave the higher mean (0.919 versus 0.910, difference 0.009).

The distribution of EQ-5D-5L index scores under each value set is shown in Supplementary Figure S1. Both distributions were left-skewed, with a mode at 1.000. The distribution under the UK value set extended further into negative values than that under the England value set.

### Socioeconomic and health gradients

Supplementary Table S3 presents weighted means by subgroup, and Supplementary Figure S2 shows mean index scores by IMD quintile. A monotonic IMD gradient was observed under all three value sets. Under the UK EQ-5D-5L value set the mean index fell from 0.919 in Q1 to 0.852 in Q5, a gap of 0.068, and under the England EQ-5D-5L value set from 0.910 to 0.852, a gap of 0.059. Because the comparison is within respondent, the difference in gradients can be tested directly: it was 0.009 (95% CI 0.006 to 0.012, *P* < 0.001). Expressed per standard deviation of the respective index, however, the two gradients were nearly identical, at 0.33 and 0.32, so the larger absolute gradient under the UK value set follows substantially from its wider dispersion. 

Apart from self-rated general health, limiting longstanding illness was associated with the largest unadjusted absolute differences in mean index scores: 0.270 under the UK EQ-5D-5L value set and 0.258 under the England EQ-5D-5L value set. A monotonic gradient was also observed across categories of self-rated general health under all three value sets (Supplementary Table S3).

### Ceiling effect by age

**Fig. 2:**
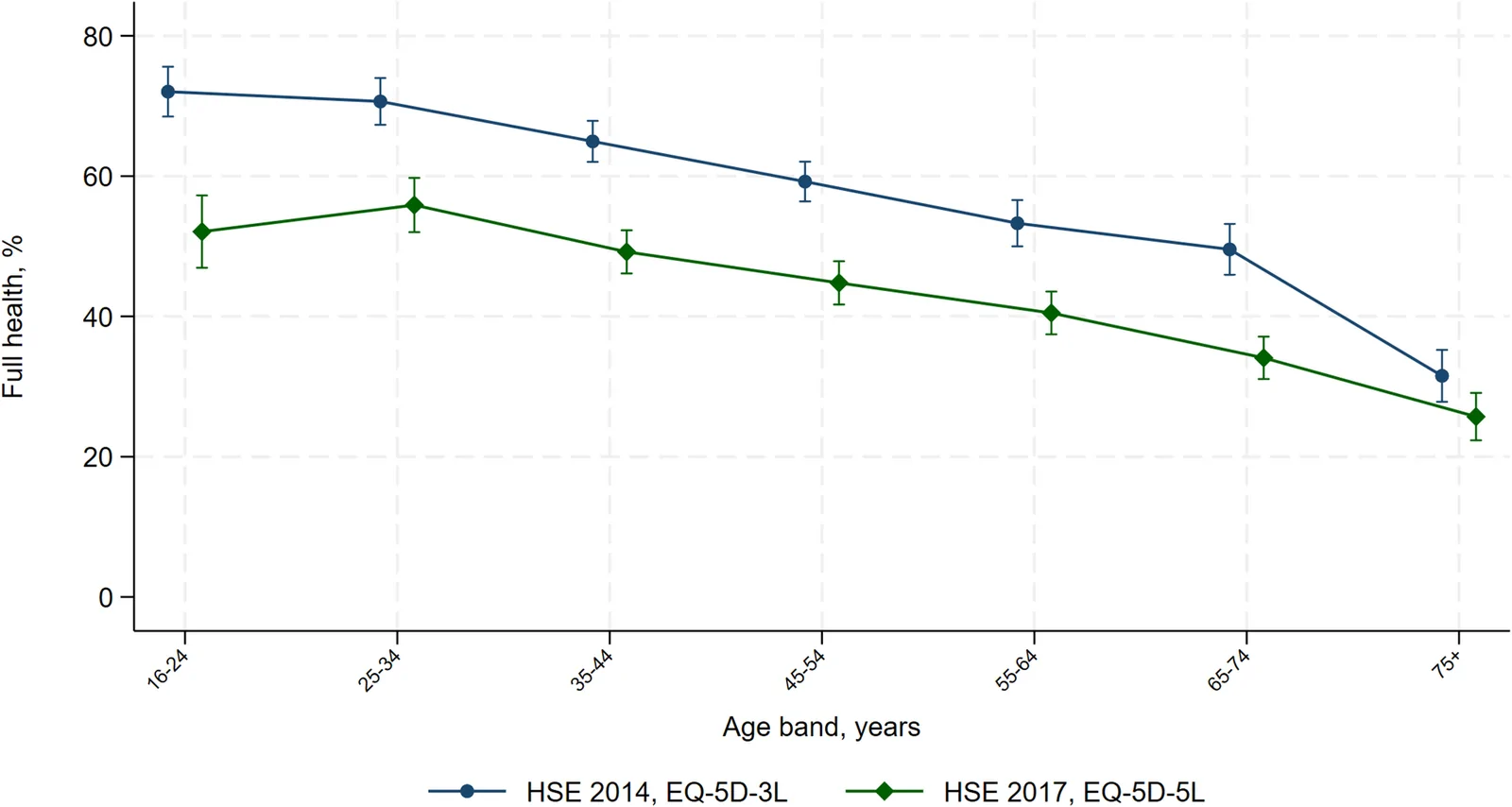
Proportion reporting full health (profile 11111) by age group, EQ-5D-3L compared with EQ-5D-5L. Full health is defined by the response profile 11111 and is a property of the descriptive system rather than of any value set. It is the exact complement of the proportion reporting any problem, which is therefore not shown. Rates are survey-weighted. Both waves use the standard Health Survey for England ten-year age banding. EQ-5D-3L data are from Health Survey for England 2014 (n = 7,085) and EQ-5D-5L data from Health Survey for England 2017 (n = 7,169); the two are independent cross-sectional samples. Error bars are 95% confidence intervals from survey-weighted estimates

Figure 2 presents the ceiling effect by age group for both instruments. The ceiling effect generally declined with age and was lower under the EQ-5D-5L than under the EQ-5D-3L in every age stratum. The difference was 20 percentage points among respondents aged 16–24 years, 13–16 percentage points across age groups from 25 to 74 years, and 6 percentage points among respondents aged ≥75 years. We do not show the proportion reporting any problem separately because it is the exact complement of the ceiling. Dimension-level response frequencies are reported in Supplementary Table S4, and the 20 most frequent EQ-5D-3L and EQ-5D-5L health-state profiles are reported in Supplementary Tables S5a and S5b, respectively.

### Adjusted associations

Survey-weighted regression results for all index outcomes and both EQ VAS outcomes are reported in Supplementary Table S6. Age, deprivation and limiting longstanding illness were each independently associated with every reported outcome in the fully adjusted models (Supplementary Table S6). Limiting longstanding illness had adjusted coefficients of −0.294 under the UK EQ-5D-3L value set, −0.255 under the UK EQ-5D-5L value set and −0.243 under the England EQ-5D-5L value set on the index scale, all with *P* < 0.001. Expressed per standard deviation of the respective index these correspond to 1.32, 1.24 and 1.30 standard deviations. It was also associated with EQ VAS scores 20.0 points lower in HSE 2014 and 19.8 points lower in HSE 2017, both with P<0.001.

The adjusted IMD gradient was also observed across outcomes. Respondents in the most deprived quintile had index scores that were 0.046 lower under the UK EQ-5D-3L value set, 0.045 lower under the UK EQ-5D-5L value set and 0.039 lower under the England EQ-5D-5L value set than those in the least deprived quintile, all with *P* < 0.001, corresponding to 0.21, 0.22 and 0.21 standard deviations. EQ VAS scores were 3.9 points lower in HSE 2014 and 4.4 points lower in HSE 2017 among respondents in the most deprived quintile compared with those in the least deprived quintile, both with *P* < 0.001.

The pattern of statistically significant covariates was similar across the two EQ-5D-5L value sets. 

### Sensitivity analyses

Tobit estimates accounting for the ceiling at 1 are reported in Supplementary Table S7. The direction and statistical significance of the key associations were unchanged compared with the primary ordinary least squares models. Absolute coefficients were larger in the Tobit models than in the ordinary least squares models. The EQ VAS generalised linear model is reported in Supplementary Table S8. The direction and statistical significance of the associations with deprivation, age, and limiting longstanding illness were consistent with the ordinary least squares findings.

## Discussion

This study provides updated EQ-5D population norms for community-dwelling adults in England using two waves of the Health Survey for England and, to our knowledge, the first set of England norms for the EQ-5D-5L scored with the new UK EQ-5D-5L value set [26]. The UK and England EQ-5D-5L value sets produced equivalent population-level estimates against a prespecified margin of 0.030, but diverged for severe health states. These findings suggest that existing English general-population estimates based on the England value set remain broadly interpretable at the population level, but analyses involving severe morbidity or health inequalities may be more sensitive to the transition to the new UK value set.

The most policy-relevant implication concerns the lower tail of the EQ-5D-5L distribution. The two value sets agreed closely for most respondents, but the UK value set produced lower scores among those reporting very poor health and extended further into negative values. Decomposition of the difference by dimension helps explain this pattern: among respondents with no dimension at level 4 or worse the UK value set scored 0.013 higher, driven by anxiety or depression and by pain, whereas among those with any dimension at level 4 or worse it scored 0.060 lower, with pain alone contributing −0.054 (Supplementary Table S9). This pattern is consistent with differences in the underlying valuation studies, including differences in dimension weights between the two value sets [12, 26] and the different elicitation and modelling approach used in the England valuation study, which combined composite TTO and discrete choice experiment data [12, 13]. For HTA, the choice of value set may have little effect on population-level estimates dominated by mild or moderate health states but may matter in evaluations where patients spend substantial time in severe health states. In such settings, estimated quality-adjusted life-year gains may differ depending on whether health improvements are scored using the UK or the England EQ-5D-5L value set. This is consistent with previous evidence that switching between EQ-5D versions or value sets can alter incremental cost-effectiveness ratios in clinically meaningful ways [38, 39]. To facilitate comparison across index scales, we also expressed adjusted between-group differences relative to the standard deviation of each index. Minimally important differences are not a suitable benchmark for them: they are thresholds for within-person change, and the reported band varies with treatment type and narrows as baseline score rises [40].

The socioeconomic findings are also important. We observed a clear deprivation gradient across all value sets, but the absolute gradient was larger when EQ-5D-5L responses were scored using the UK value set than the England value set. This indicates that value-set choice can influence not only mean population utility estimates, but also the apparent magnitude of health inequality. Because EQ-5D norms are used as background utilities in economic models, comparators for patient populations, and summary measures in population health monitoring, a larger absolute deprivation gradient under the UK value set may have implications for equity-focused analyses, although relative to their respective standard deviations, the two value sets gave nearly identical gradients. Researchers using EQ-5D-5L data in England should therefore report the value set used and consider whether conclusions about socioeconomic inequality are robust to alternative scoring where appropriate.

The comparison with the EQ-5D-3L provides useful context for the transition in national survey measurement, but should not be interpreted as a direct comparison of instruments or value sets. HSE 2014 and HSE 2017 are independent cross-sectional samples. With both waves placed on the UK EQ-5D-3L scale via the crosswalk, the wave difference was −0.029, of which age and sex composition explained 7.7% and sociodemographic covariates 1.8% (Supplementary Table S10). Composition therefore accounts for very little of it; the residual combines period change with instrument version and cannot be apportioned between them, because no respondent completed both instruments. Nevertheless, the descriptive findings are consistent with previous evidence that the EQ-5D-5L reduces ceiling effects and captures more reported problems than the EQ-5D-3L [4, 5, 41]. This matters for population norms because ceiling effects can compress variation, particularly among younger or healthier respondents, and may limit the ability of the EQ-5D-3L to detect differences across sociodemographic groups or over time. The inclusion of the EQ VAS provides a useful value-set-independent benchmark, although it should be interpreted as a self-rated health measure rather than a preference-based utility [42].

Our findings align with previous UK and international population norms studies showing lower EQ-5D index scores with increasing age, lower scores among women, and substantial decrements associated with limiting longstanding illness [6, 8, 9]. The EQ-5D-5L estimates for England also sit within the range reported in norms studies from other countries, including the United States [43], Germany [44], Japan [45], Singapore [46], Peninsular Malaysia [47], and multinational comparisons [48, 49]. The present study adds to this literature by showing how the same EQ-5D-5L descriptive responses generate different population norms under the UK and England value sets. This distinction is important because population norms are not purely descriptive properties of a sample; they also reflect the value set used to convert health states into utilities. Published norms should therefore be interpreted as instrument- and value-set-specific reference values rather than universal estimates of population health.

This study has several strengths. We used large, nationally representative HSE samples and incorporated survey weights, strata, and primary sampling units in the primary analyses. EQ-5D-5L responses from HSE 2017 were scored in parallel using both the UK and England EQ-5D-5L value sets, allowing direct within-respondent comparison without confounding by sample composition. We reported the EQ VAS alongside index scores and examined both unadjusted norms and adjusted associations. Sensitivity analyses using upper-censored Tobit models for the EQ-5D index and a fractional-response generalised linear model for the EQ VAS supported the direction and statistical significance of the main findings. Several limitations should be considered. First, HSE 2014 and HSE 2017 were selected because they bracketed the transition from EQ-5D-3L to EQ-5D-5L in the HSE [9, 33, 34]. They are not longitudinally linked, and differences between 3L and 5L estimates may reflect instrument version, value-set scaling, sample composition, and secular changes in population health. We therefore restricted formal value-set comparisons to HSE 2017. Second, the EQ-5D-5L data predate the COVID-19 pandemic and should be interpreted as pre-pandemic norms. Updated population norms using post-pandemic EQ-5D-5L data will be needed when suitable data become available [50]. Third, the HSE samples people living in private households and excludes communal establishments, including care homes, which may lead to overestimation of population mean utilities at older ages [11]. Fourth, ethnicity was collapsed into a binary variable because of small numbers in some groups, which precluded more detailed analysis of ethnic inequalities. Finally, alternative regression models cannot fully accommodate all features of the bounded, multimodal EQ-5D distribution. Although the Tobit and generalised linear model sensitivity analyses incorporated the full HSE complex survey design, they should be interpreted as supportive rather than as the basis for primary inference.

In conclusion, the UK and England EQ-5D-5L value sets produce equivalent EQ-5D-5L norms for community-dwelling adults in England at the population level, but diverge for severe health states. With the UK EQ-5D-5L value set adopted for reference-case analyses in adults from 27 August 2026, these norms provide reference values on the scale now recommended for NICE reference-case analyses and quantify differences relative to the former NICE mapping approach. These differences are unlikely to affect all applications equally. They matter most where analyses involve severe morbidity, inequality measurement, or comparisons across evidence generated using different value sets. The norms reported here provide reference values for HTA, comparative effectiveness research, equity analysis, and population health monitoring as EQ-5D-5L scoring practice in the United Kingdom transitions to the new UK value set.

## Supplementary Information

Supplementary Material 1

## Data Availability

This study used publicly available, fully anonymised data from the Health Survey for England 2014 and 2017, accessed via the UK Data Service (SN 7919 and SN 8488). Stata analysis code is available from the corresponding author upon reasonable request.
