# Supplementary material for: EQ-5D population norms for England: comparing the UK and England EQ-5D-5L value sets, the former NICE reference-case mapping and the crosswalk
Source: Qual Life Res. 2026 Nov 6;35(11):319. doi: 10.1007/s11136-026-04417-3 (PMC13743634; doi:10.1007/s11136-026-04417-3)
Supplement: Supplementary file 1 [file 11136_2026_4417_MOESM1_ESM.docx]

**Table S1** Derivation of the analytic samples and survey design diagnostics, Health Survey for England 2014 and 2017

| **Step** | **HSE 2014** | **HSE 2017** |
| --- | --- | --- |
| **Panel A. Derivation of the analytic sample** | | |
| Step | HSE 2014 | HSE 2017 |
| Records in the raw UKDA file, all ages | 10,080 | 9,982 |
| Aged 16 years and over | 8,077 | 7,997 |
| Excluded: incomplete EQ-5D profile | 992 | 828 |
| With a complete EQ-5D profile | 7,085 | 7,169 |
| Excluded: missing or zero interview weight | 0 | 0 |
| ANALYTIC SAMPLE | 7,085 | 7,169 |
| **Panel B. Weight and design diagnostics** | | |
| Quantity | HSE 2014 | HSE 2017 |
| Weight variable | wt_int (core interview weight) | wt_int (core interview weight) |
| Strata | 158 | 201 |
| Primary sampling units | 561 | 530 |
| Mean weight | 1.010 | 0.996 |
| Median weight | 0.908 | 0.858 |
| Weight range | 0.340 to 6.288 | 0.319 to 6.548 |
| Coefficient of variation of the weights | 0.422 | 0.488 |
| Kish effective sample size | 6015 | 5789 |
| Design effect from weighting (n divided by effective n) | 1.178 | 1.238 |

The Health Survey for England is an annual cross-sectional survey of the general population living in private households in England. It uses a stratified multi-stage probability design: postcode sectors are selected with probability proportional to size within strata defined by region and deprivation, addresses are then selected systematically within each, and all adults aged 16 years and over at a selected address are eligible

The core interview weight is applied throughout. It corrects for the unequal probability of selection arising from the address-based design, for household size and for non-response, and is calibrated to Office for National Statistics population estimates by age, sex and region. It is the appropriate weight because the EQ-5D is administered within the core interview rather than at the nurse visit

All estimates apply the weight together with the primary sampling units and strata, using Taylor linearisation. Strata containing a single sampling unit are treated as certainty units, and confidence intervals use the design degrees of freedom

The design effect shown reflects variation in the weights only, following the Kish approximation. It does not include the clustering component, which is captured in the linearised standard errors reported throughout

HSE Health Survey for England

**Table S2** Value set coefficients and provenance of the two conversion routes

| **Term** | **Decrement** |  |  |
| --- | --- | --- | --- |
| **Panel A. UK EQ-5D-3L value set (Dolan 1997)** | | | |
| Term | Decrement |  |  |
| Constant (any problem) | 0.081 |  |  |
| Mobility, level 2 | 0.069 |  |  |
| Mobility, level 3 | 0.314 |  |  |
| Self-care, level 2 | 0.104 |  |  |
| Self-care, level 3 | 0.214 |  |  |
| Usual activities, level 2 | 0.036 |  |  |
| Usual activities, level 3 | 0.094 |  |  |
| Pain or discomfort, level 2 | 0.123 |  |  |
| Pain or discomfort, level 3 | 0.386 |  |  |
| Anxiety or depression, level 2 | 0.071 |  |  |
| Anxiety or depression, level 3 | 0.236 |  |  |
| N3 (any dimension at level 3) | 0.269 |  |  |
| Worst state 33333, computed from the above | −0.594 |  |  |
| **Panel B. EQ-5D-5L value sets, decrements by dimension and level** | | | |
| Dimension | Level | UK EQ-5D-5L | England EQ-5D-5L |
| Mobility | 2 | 0.032 | 0.058 |
|  | 3 | 0.058 | 0.076 |
|  | 4 | 0.179 | 0.207 |
|  | 5 | 0.279 | 0.274 |
| Self-care | 2 | 0.038 | 0.050 |
|  | 3 | 0.060 | 0.080 |
|  | 4 | 0.162 | 0.164 |
|  | 5 | 0.206 | 0.203 |
| Usual activities | 2 | 0.049 | 0.050 |
|  | 3 | 0.086 | 0.063 |
|  | 4 | 0.184 | 0.162 |
|  | 5 | 0.212 | 0.184 |
| Pain or discomfort | 2 | 0.056 | 0.063 |
|  | 3 | 0.066 | 0.084 |
|  | 4 | 0.371 | 0.276 |
|  | 5 | 0.479 | 0.335 |
| Anxiety or depression | 2 | 0.041 | 0.078 |
|  | 3 | 0.126 | 0.104 |
|  | 4 | 0.313 | 0.285 |
|  | 5 | 0.391 | 0.289 |
| Worst state 55555, computed from the above |  | −0.567 | −0.285 |
| **Panel C. Routes that are not coefficient-based** | | | |
| Route | Description |  |  |
| UK EQ-5D-3L (mapped) | EQ-5D-5L responses mapped to the 1997 UK EQ-5D-3L value set with the NICE Decision Support Unit command eq5dmap, version 3.01 (24 February 2026), direction 5->3, target value set UK, predicting from the five descriptive items. The model is a bivariate ordered specification per dimension with a copula linking the two latent variables, conditional on age band and sex. The command returns the expectation of the 3L index over the implied distribution of 3L responses, so it is a fitted value rather than a tariff. No cell of the lookup table reaches 1.000; the maximum is 0.9885 and the minimum is −0.5316. | | |
| UK EQ-5D-3L (crosswalk) | EQ-5D-5L responses converted to the 1997 UK EQ-5D-3L value set with the crosswalk of van Hout et al. (2012), a deterministic lookup over all 3,125 EQ-5D-5L states obtained from the EuroQol EQ-5D Suite. Profile 11111 returns 1.000 and profile 55555 returns −0.594, the 1997 worst-state value. | | |

Coefficients are transcribed from the published sources. Each index is one minus the sum of the applicable decrements. The UK EQ-5D-3L value set additionally applies a constant where any problem is reported and an N3 term where any dimension is rated level 3

Worst-state values are computed from the coefficients printed above rather than quoted, and serve as a check on transcription. They should read −0.594, −0.567 and −0.285 respectively

Value sets: UK EQ-5D-3L, Dolan 1997; UK EQ-5D-5L, Rowen et al. 2026; England EQ-5D-5L, Devlin et al. 2018. Where EQ-5D-5L responses are converted to the UK EQ-5D-3L value set, mapped denotes the NICE Decision Support Unit algorithm and crosswalk denotes the algorithm of van Hout et al. (2012)

The mapped score is a conditional expectation given the EQ-5D-5L response profile, age and sex; its maximum is 0.9885, so it has no ceiling. Its overall standard deviation, minimum and proportion below zero are reported in Table 2 of the main article. Scores on the UK EQ-5D-3L and EQ-5D-5L scales are not directly comparable in level

**Table S3** Survey-weighted mean EQ-5D index scores by subgroup, with 95% confidence intervals, Health Survey for England 2014 and 2017

|  | | | **HSE 2014** | **HSE 2017** | | | | |
| --- | --- | --- | --- | --- | --- | --- | --- | --- |
| **Subgroup** | **Category** | **n** | **UK EQ-5D-3L** | **n** | **UK EQ-5D-5L** | **England EQ-5D-5L** | **UK EQ-5D-3L (mapped)** | **UK EQ-5D-3L (crosswalk)** |
| Ethnicity | White | 6,369 | 0.865 (0.858 to 0.872) | 6,340 | 0.888 (0.882 to 0.895) | 0.883 (0.877 to 0.888) | 0.830 (0.823 to 0.837) | 0.836 (0.829 to 0.842) |
|  | Non-white | 710 | 0.878 (0.859 to 0.898) | 818 | 0.906 (0.893 to 0.920) | 0.901 (0.888 to 0.913) | 0.855 (0.841 to 0.869) | 0.853 (0.837 to 0.869) |
| Marital status | Married or cohabiting | 4,526 | 0.885 (0.878 to 0.892) | 4,700 | 0.906 (0.901 to 0.912) | 0.899 (0.894 to 0.905) | 0.850 (0.843 to 0.856) | 0.854 (0.847 to 0.860) |
|  | Single | 1,361 | 0.887 (0.875 to 0.899) | 1,308 | 0.896 (0.885 to 0.907) | 0.892 (0.882 to 0.902) | 0.845 (0.832 to 0.858) | 0.849 (0.837 to 0.862) |
|  | Separated, divorced or widowed | 1,196 | 0.754 (0.736 to 0.772) | 1,161 | 0.806 (0.789 to 0.824) | 0.803 (0.787 to 0.819) | 0.732 (0.714 to 0.751) | 0.742 (0.724 to 0.759) |
| Education | Degree or higher | 1,862 | 0.920 (0.912 to 0.928) | 2,098 | 0.928 (0.921 to 0.936) | 0.921 (0.914 to 0.927) | 0.877 (0.868 to 0.885) | 0.878 (0.869 to 0.887) |
|  | Below degree | 5,213 | 0.846 (0.839 to 0.854) | 5,058 | 0.874 (0.867 to 0.881) | 0.869 (0.863 to 0.875) | 0.814 (0.807 to 0.821) | 0.820 (0.813 to 0.827) |
| Employment status | Employed | 3,907 | 0.918 (0.912 to 0.924) | 3,892 | 0.939 (0.935 to 0.942) | 0.930 (0.926 to 0.934) | 0.886 (0.881 to 0.891) | 0.890 (0.884 to 0.895) |
|  | Retired | 1,830 | 0.787 (0.773 to 0.800) | 2,006 | 0.835 (0.823 to 0.848) | 0.828 (0.816 to 0.840) | 0.762 (0.749 to 0.776) | 0.769 (0.756 to 0.782) |
|  | Unemployed | 275 | 0.904 (0.882 to 0.925) | 115 | 0.881 (0.849 to 0.914) | 0.874 (0.844 to 0.904) | 0.816 (0.778 to 0.853) | 0.822 (0.786 to 0.859) |
|  | Other economically inactive | 1,068 | 0.779 (0.758 to 0.801) | 1,155 | 0.805 (0.785 to 0.825) | 0.810 (0.792 to 0.828) | 0.750 (0.729 to 0.771) | 0.756 (0.735 to 0.777) |
| IMD deprivation quintile | Q1 (least deprived) | 1,603 | 0.906 (0.896 to 0.916) | 1,500 | 0.919 (0.910 to 0.929) | 0.910 (0.901 to 0.919) | 0.862 (0.851 to 0.873) | 0.866 (0.855 to 0.877) |
|  | Q2 | 1,427 | 0.880 (0.868 to 0.892) | 1,552 | 0.908 (0.897 to 0.919) | 0.901 (0.890 to 0.911) | 0.852 (0.839 to 0.865) | 0.858 (0.845 to 0.870) |
|  | Q3 | 1,388 | 0.858 (0.843 to 0.873) | 1,372 | 0.901 (0.891 to 0.910) | 0.893 (0.884 to 0.903) | 0.842 (0.830 to 0.854) | 0.849 (0.837 to 0.860) |
|  | Q4 | 1,383 | 0.850 (0.836 to 0.864) | 1,406 | 0.869 (0.856 to 0.883) | 0.864 (0.852 to 0.877) | 0.810 (0.796 to 0.825) | 0.812 (0.797 to 0.827) |
|  | Q5 (most deprived) | 1,284 | 0.832 (0.817 to 0.847) | 1,339 | 0.852 (0.836 to 0.867) | 0.852 (0.838 to 0.865) | 0.794 (0.778 to 0.810) | 0.800 (0.784 to 0.815) |
| Longstanding illness | Yes | 2,920 | 0.740 (0.728 to 0.752) | 3,296 | 0.795 (0.784 to 0.806) | 0.792 (0.782 to 0.802) | 0.717 (0.706 to 0.728) | 0.726 (0.715 to 0.737) |
|  | No | 4,160 | 0.946 (0.942 to 0.949) | 3,869 | 0.962 (0.959 to 0.965) | 0.954 (0.951 to 0.957) | 0.919 (0.915 to 0.923) | 0.921 (0.916 to 0.926) |
| Limiting longstanding illness | Yes | 1,663 | 0.617 (0.601 to 0.633) | 1,895 | 0.686 (0.669 to 0.702) | 0.689 (0.675 to 0.703) | 0.595 (0.579 to 0.610) | 0.609 (0.594 to 0.624) |
|  | No | 5,417 | 0.935 (0.931 to 0.938) | 5,270 | 0.956 (0.953 to 0.958) | 0.947 (0.944 to 0.950) | 0.909 (0.905 to 0.912) | 0.910 (0.906 to 0.914) |
| Self-rated general health | Very good | 2,339 | 0.963 (0.959 to 0.967) | 2,353 | 0.968 (0.965 to 0.972) | 0.962 (0.959 to 0.966) | 0.931 (0.926 to 0.935) | 0.935 (0.930 to 0.940) |
|  | Good | 3,026 | 0.903 (0.897 to 0.908) | 2,975 | 0.935 (0.931 to 0.939) | 0.924 (0.920 to 0.928) | 0.876 (0.871 to 0.882) | 0.876 (0.871 to 0.882) |
|  | Fair | 1,229 | 0.747 (0.731 to 0.763) | 1,272 | 0.811 (0.800 to 0.823) | 0.800 (0.789 to 0.811) | 0.720 (0.707 to 0.733) | 0.725 (0.712 to 0.739) |
|  | Bad | 371 | 0.449 (0.416 to 0.482) | 408 | 0.501 (0.463 to 0.540) | 0.529 (0.496 to 0.561) | 0.419 (0.383 to 0.455) | 0.449 (0.415 to 0.483) |
|  | Very bad | 118 | 0.248 (0.190 to 0.306) | 159 | 0.243 (0.172 to 0.313) | 0.313 (0.255 to 0.371) | 0.197 (0.134 to 0.260) | 0.231 (0.169 to 0.294) |

Cells show the survey-weighted mean and its 95% confidence interval, to three decimal places

Value sets: UK EQ-5D-3L, Dolan 1997; UK EQ-5D-5L, Rowen et al. 2026; England EQ-5D-5L, Devlin et al. 2018. Where EQ-5D-5L responses are converted to the UK EQ-5D-3L value set, mapped denotes the NICE Decision Support Unit algorithm and crosswalk denotes the algorithm of van Hout et al. (2012)

The mapped score is a conditional expectation given the EQ-5D-5L response profile, age and sex; its maximum is 0.9885, so it has no ceiling. Its overall standard deviation, minimum and proportion below zero are reported in Table 2 of the main article. Scores on the UK EQ-5D-3L and EQ-5D-5L scales are not directly comparable in level

HSE Health Survey for England, IMD Index of Multiple Deprivation

**Table S4** Survey-weighted distribution of EQ-5D dimension responses, Health Survey for England 2014 and 2017

| **Dimension** | **Level 1, %** | **Level 2, %** | **Level 3, %** | **Level 4, %** | **Level 5, %** | **Any problem, %** | **Level 4 or worse, %** |
| --- | --- | --- | --- | --- | --- | --- | --- |
| **HSE 2014, EQ-5D-3L** | | | | | | | |
| Dimension | Level 1, % | Level 2, % | Level 3, % | Any problem, % |  |  |  |
| Mobility | 84.5 | 15.4 | 0.2 | 15.5 |  |  |  |
| Self-care | 95.2 | 4.5 | 0.3 | 4.8 |  |  |  |
| Usual activities | 85.6 | 13.1 | 1.3 | 14.4 |  |  |  |
| Pain or discomfort | 69.8 | 26.8 | 3.4 | 30.2 |  |  |  |
| Anxiety or depression | 81.1 | 16.8 | 2.0 | 18.9 |  |  |  |
| **HSE 2017, EQ-5D-5L** | | | | | | | |
| Dimension | Level 1, % | Level 2, % | Level 3, % | Level 4, % | Level 5, % | Any problem, % | Level 4 or worse, % |
| Mobility | 79.1 | 10.5 | 5.7 | 4.2 | 0.6 | 20.9 | 4.8 |
| Self-care | 92.5 | 3.7 | 2.6 | 1.0 | 0.3 | 7.5 | 1.3 |
| Usual activities | 80.3 | 10.4 | 5.6 | 2.8 | 0.9 | 19.7 | 3.7 |
| Pain or discomfort | 57.6 | 25.6 | 11.7 | 4.0 | 1.2 | 42.4 | 5.2 |
| Anxiety or depression | 69.6 | 18.7 | 8.4 | 2.5 | 0.8 | 30.4 | 3.3 |

Percentages are survey-weighted and estimated among respondents with a non-missing response on that dimension. Rows sum to 100 across the level columns, subject to rounding

Dimension-level any-problem proportions are retained because, unlike the overall any-problem proportion, they are not the complement of a quantity reported elsewhere; they underpin the decomposition in Table S9

The two descriptive systems have different numbers of levels and different level wording, so columns are not comparable between the two panels. EQ-5D-3L level 3 is the most severe level on each dimension, whereas EQ-5D-5L level 3 is the middle level

HSE Health Survey for England

**Table S5a** Twenty most frequently reported EQ-5D-3L health states, Health Survey for England 2014

| **Rank** | **Profile** | **n** | **Unweighted %** | **Weighted %** | **UK EQ-5D-3L** |
| --- | --- | --- | --- | --- | --- |
| 1 | 11111 | 4,069 | 57.43 | 59.64 | 1.000 |
| 2 | 11121 | 769 | 10.85 | 10.25 | 0.796 |
| 3 | 11112 | 465 | 6.56 | 7.08 | 0.848 |
| 4 | 11122 | 232 | 3.27 | 3.09 | 0.725 |
| 5 | 21221 | 229 | 3.23 | 2.93 | 0.691 |
| 6 | 21121 | 214 | 3.02 | 2.75 | 0.727 |
| 7 | 21222 | 123 | 1.74 | 1.51 | 0.620 |
| 8 | 11221 | 90 | 1.27 | 1.14 | 0.760 |
| 9 | 22221 | 72 | 1.02 | 0.89 | 0.587 |
| 10 | 22222 | 66 | 0.93 | 0.86 | 0.516 |
| 11 | 21111 | 60 | 0.85 | 0.74 | 0.850 |
| 12 | 11222 | 56 | 0.79 | 0.85 | 0.689 |
| 13 | 22232 | 52 | 0.73 | 0.62 | −0.016 |
| 14 | 21122 | 50 | 0.71 | 0.65 | 0.656 |
| 15 | 21232 | 39 | 0.55 | 0.53 | 0.088 |
| 16 | 21231 | 35 | 0.49 | 0.45 | 0.159 |
| 17 | 21211 | 31 | 0.44 | 0.39 | 0.814 |
| 18 | 22231 | 31 | 0.44 | 0.34 | 0.055 |
| 19 | 11212 | 24 | 0.34 | 0.38 | 0.812 |
| 20 | 11211 | 20 | 0.28 | 0.27 | 0.883 |

Profiles are ordered by unweighted frequency. Ninety-four distinct EQ-5D-3L states were observed out of 243 possible states. Percentages are of the analytic sample

Value sets: UK EQ-5D-3L, Dolan 1997

**Table S5b** Twenty most frequently reported EQ-5D-5L health states, Health Survey for England 2017, scored four ways

| **Rank** | **Profile** | **n** | **Unweighted %** | **Weighted %** | **UK EQ-5D-5L** | **England EQ-5D-5L** | **UK EQ-5D-3L (mapped)** | **UK EQ-5D-3L (crosswalk)** |
| --- | --- | --- | --- | --- | --- | --- | --- | --- |
| 1 | 11111 | 3,045 | 42.47 | 44.61 | 1.000 | 1.000 | 0.987 | 1.000 |
| 2 | 11121 | 865 | 12.07 | 11.31 | 0.944 | 0.937 | 0.866 | 0.837 |
| 3 | 11112 | 461 | 6.43 | 7.38 | 0.959 | 0.922 | 0.891 | 0.879 |
| 4 | 11122 | 286 | 3.99 | 4.15 | 0.903 | 0.859 | 0.795 | 0.767 |
| 5 | 11113 | 165 | 2.30 | 2.55 | 0.874 | 0.896 | 0.815 | 0.848 |
| 6 | 21121 | 151 | 2.11 | 2.09 | 0.912 | 0.879 | 0.799 | 0.767 |
| 7 | 11131 | 122 | 1.70 | 1.43 | 0.934 | 0.916 | 0.782 | 0.796 |
| 8 | 21221 | 104 | 1.45 | 1.37 | 0.863 | 0.829 | 0.744 | 0.735 |
| 9 | 11123 | 75 | 1.05 | 1.11 | 0.818 | 0.833 | 0.737 | 0.749 |
| 10 | 11221 | 64 | 0.89 | 0.87 | 0.895 | 0.887 | 0.807 | 0.795 |
| 11 | 21231 | 64 | 0.89 | 0.76 | 0.853 | 0.808 | 0.672 | 0.710 |
| 12 | 11132 | 52 | 0.73 | 0.68 | 0.893 | 0.838 | 0.725 | 0.740 |
| 13 | 21222 | 52 | 0.73 | 0.63 | 0.822 | 0.751 | 0.697 | 0.679 |
| 14 | 21131 | 47 | 0.66 | 0.57 | 0.902 | 0.858 | 0.719 | 0.739 |
| 15 | 21111 | 42 | 0.59 | 0.57 | 0.968 | 0.942 | 0.914 | 0.877 |
| 16 | 21122 | 40 | 0.56 | 0.54 | 0.871 | 0.801 | 0.746 | 0.708 |
| 17 | 11222 | 39 | 0.54 | 0.49 | 0.854 | 0.809 | 0.754 | 0.736 |
| 18 | 21232 | 39 | 0.54 | 0.46 | 0.812 | 0.730 | 0.630 | 0.654 |
| 19 | 31231 | 37 | 0.52 | 0.42 | 0.827 | 0.790 | 0.651 | 0.698 |
| 20 | 31331 | 29 | 0.40 | 0.38 | 0.790 | 0.777 | 0.628 | 0.691 |

Profiles are ordered by unweighted frequency. Four hundred and eighty-one distinct EQ-5D-5L states were observed out of 3,125 possible states. Percentages are of the analytic sample

The mapped column is the mean of a conditional expectation within each profile and therefore varies with the age and sex composition of the respondents reporting it. Unlike the other three columns it is not a fixed value attached to the profile

Value sets: UK EQ-5D-3L, Dolan 1997; UK EQ-5D-5L, Rowen et al. 2026; England EQ-5D-5L, Devlin et al. 2018. Where EQ-5D-5L responses are converted to the UK EQ-5D-3L value set, mapped denotes the NICE Decision Support Unit algorithm and crosswalk denotes the algorithm of van Hout et al. (2012)

The mapped score does not reach 1.000 (maximum 0.9885) and therefore has no ceiling. Its overall dispersion and range are reported in Table 2 of the main article

**Table S6** Adjusted associations with EQ-5D index scores and the EQ VAS: survey-weighted linear regression, Health Survey for England 2014 and 2017

| **Covariate** | **UK EQ-5D-3L, 2014** | **UK EQ-5D-5L, 2017** | **England EQ-5D-5L, 2017** | **Mapped, 2017** | **Crosswalk, 2017** | **EQ VAS, 2014** | **EQ VAS, 2017** |
| --- | --- | --- | --- | --- | --- | --- | --- |
| Age band 2 vs band 1 | **−0.019 (−0.035 to −0.004)** | −0.007 (−0.023 to 0.009) | −0.003 (−0.018 to 0.011) | 0.000 (−0.017 to 0.017) | −0.000 (−0.018 to 0.018) | **−2.092 (−3.944 to −0.239)** | −0.219 (−2.316 to 1.877) |
| Age band 3 vs band 1 | **−0.040 (−0.058 to −0.022)** | **−0.024 (−0.042 to −0.007)** | **−0.021 (−0.037 to −0.006)** | **−0.026 (−0.044 to −0.008)** | **−0.027 (−0.045 to −0.008)** | −1.293 (−3.206 to 0.620) | −0.691 (−2.757 to 1.375) |
| Age band 4 vs band 1 | **−0.042 (−0.061 to −0.023)** | **−0.040 (−0.058 to −0.022)** | **−0.035 (−0.051 to −0.020)** | **−0.046 (−0.064 to −0.027)** | **−0.044 (−0.063 to −0.026)** | −1.883 (−3.862 to 0.096) | −1.375 (−3.459 to 0.710) |
| Age band 5 vs band 1 | **−0.068 (−0.087 to −0.048)** | **−0.038 (−0.056 to −0.020)** | **−0.036 (−0.052 to −0.020)** | **−0.051 (−0.070 to −0.032)** | **−0.048 (−0.067 to −0.029)** | **−2.975 (−5.160 to −0.789)** | −0.617 (−2.779 to 1.544) |
| Age band 6 vs band 1 | **−0.049 (−0.068 to −0.029)** | **−0.033 (−0.052 to −0.014)** | **−0.033 (−0.050 to −0.016)** | **−0.045 (−0.065 to −0.025)** | **−0.048 (−0.068 to −0.027)** | −1.476 (−3.484 to 0.532) | 0.328 (−1.848 to 2.505) |
| Age band 7 vs band 1 | **−0.060 (−0.083 to −0.036)** | **−0.041 (−0.063 to −0.018)** | **−0.046 (−0.066 to −0.026)** | **−0.056 (−0.079 to −0.033)** | **−0.059 (−0.082 to −0.035)** | **−4.918 (−7.269 to −2.567)** | **−2.812 (−5.205 to −0.418)** |
| Female vs male | **−0.013 (−0.022 to −0.004)** | **−0.012 (−0.021 to −0.004)** | **−0.012 (−0.020 to −0.004)** | **−0.020 (−0.029 to −0.011)** | **−0.016 (−0.025 to −0.007)** | 0.149 (−0.656 to 0.953) | −0.793 (−1.619 to 0.033) |
| White vs non-white | 0.006 (−0.010 to 0.022) | 0.000 (−0.013 to 0.013) | 0.001 (−0.011 to 0.013) | −0.001 (−0.015 to 0.013) | 0.006 (−0.010 to 0.021) | **2.604 (1.065 to 4.142)** | 1.556 (−0.065 to 3.178) |
| Single vs married or cohabiting | **−0.025 (−0.040 to −0.011)** | **−0.015 (−0.028 to −0.002)** | **−0.012 (−0.023 to −0.001)** | −0.013 (−0.026 to 0.001) | −0.012 (−0.026 to 0.002) | **−1.730 (−3.189 to −0.272)** | −1.332 (−2.677 to 0.013) |
| Separated, divorced or widowed vs married or cohabiting | **−0.054 (−0.069 to −0.038)** | **−0.038 (−0.054 to −0.022)** | **−0.035 (−0.049 to −0.021)** | **−0.041 (−0.057 to −0.026)** | **−0.039 (−0.054 to −0.023)** | **−2.836 (−4.129 to −1.543)** | **−3.058 (−4.486 to −1.629)** |
| Degree or higher vs below degree | **0.026 (0.017 to 0.035)** | **0.012 (0.003 to 0.021)** | **0.010 (0.002 to 0.019)** | **0.012 (0.002 to 0.022)** | 0.009 (−0.001 to 0.019) | 0.802 (−0.088 to 1.692) | 0.658 (−0.270 to 1.587) |
| IMD Q2 vs Q1 | **−0.014 (−0.026 to −0.003)** | −0.011 (−0.022 to 0.000) | −0.009 (−0.020 to 0.001) | −0.010 (−0.023 to 0.002) | −0.009 (−0.021 to 0.004) | −0.438 (−1.641 to 0.764) | −1.005 (−2.279 to 0.270) |
| IMD Q3 vs Q1 | **−0.021 (−0.035 to −0.008)** | **−0.012 (−0.023 to −0.001)** | **−0.011 (−0.021 to −0.001)** | **−0.013 (−0.026 to −0.001)** | −0.011 (−0.024 to 0.001) | **−1.439 (−2.697 to −0.181)** | −1.216 (−2.575 to 0.142) |
| IMD Q4 vs Q1 | **−0.030 (−0.042 to −0.017)** | **−0.036 (−0.049 to −0.024)** | **−0.034 (−0.045 to −0.023)** | **−0.039 (−0.052 to −0.025)** | **−0.041 (−0.055 to −0.027)** | **−3.173 (−4.550 to −1.796)** | **−2.494 (−3.795 to −1.194)** |
| IMD Q5 vs Q1 | **−0.046 (−0.060 to −0.032)** | **−0.045 (−0.059 to −0.031)** | **−0.039 (−0.051 to −0.026)** | **−0.044 (−0.058 to −0.030)** | **−0.044 (−0.059 to −0.030)** | **−3.906 (−5.371 to −2.442)** | **−4.396 (−5.794 to −2.998)** |
| Limiting longstanding illness vs none | **−0.294 (−0.310 to −0.277)** | **−0.255 (−0.272 to −0.239)** | **−0.243 (−0.258 to −0.229)** | **−0.295 (−0.311 to −0.279)** | **−0.283 (−0.298 to −0.267)** | **−19.981 (−21.355 to −18.607)** | **−19.823 (−21.063 to −18.584)** |
| Constant | **0.997 (0.973 to 1.020)** | **1.009 (0.990 to 1.028)** | **0.995 (0.977 to 1.013)** | **0.971 (0.950 to 0.992)** | **0.965 (0.943 to 0.988)** | **84.405 (82.143 to 86.667)** | **85.980 (83.239 to 88.722)** |

Coefficients whose 95% confidence interval excludes zero are shown in bold. This is a reading aid only and is not a claim about the importance of an association; the interval itself, not the bolding, carries the inferential content

Cells show the coefficient and its 95% confidence interval from a survey-weighted linear regression on the complete-case sample. Index coefficients are given to three decimal places; EQ VAS coefficients are on the 0 to 100 scale

Adjusted R-squared is not reported. It is not comparable across dependent variables with different ranges and variances. Coefficients expressed per standard deviation of their own outcome, which are comparable, are reported in the main text

Age enters as a categorical term. The Health Survey for England records age in bands for adults, so a continuous age coefficient would be an artefact of the banding

Value sets: UK EQ-5D-3L, Dolan 1997; UK EQ-5D-5L, Rowen et al. 2026; England EQ-5D-5L, Devlin et al. 2018. Where EQ-5D-5L responses are converted to the UK EQ-5D-3L value set, mapped denotes the NICE Decision Support Unit algorithm and crosswalk denotes the algorithm of van Hout et al. (2012)

The mapped score is a conditional expectation given the EQ-5D-5L response profile, age and sex; its maximum is 0.9885, so it has no ceiling. Its overall standard deviation, minimum and proportion below zero are reported in Table 2 of the main article. Scores on the UK EQ-5D-3L and EQ-5D-5L scales are not directly comparable in level

IMD Index of Multiple Deprivation

**Table S7** Sensitivity analysis: survey-weighted Tobit regression, upper-censored at 1, Health Survey for England 2014 and 2017

| **Covariate** | **UK EQ-5D-3L, 2014** | **UK EQ-5D-5L, 2017** | **England EQ-5D-5L, 2017** |
| --- | --- | --- | --- |
| Age band 2 vs band 1 | −0.044 (−0.090 to 0.002) | 0.005 (−0.028 to 0.038) | 0.008 (−0.023 to 0.038) |
| Age band 3 vs band 1 | **−0.099 (−0.143 to −0.054)** | **−0.039 (−0.070 to −0.008)** | **−0.033 (−0.062 to −0.005)** |
| Age band 4 vs band 1 | **−0.114 (−0.160 to −0.067)** | **−0.065 (−0.096 to −0.034)** | **−0.057 (−0.085 to −0.028)** |
| Age band 5 vs band 1 | **−0.154 (−0.197 to −0.111)** | **−0.067 (−0.098 to −0.035)** | **−0.061 (−0.090 to −0.032)** |
| Age band 6 vs band 1 | **−0.132 (−0.179 to −0.085)** | **−0.071 (−0.104 to −0.038)** | **−0.066 (−0.097 to −0.036)** |
| Age band 7 vs band 1 | **−0.165 (−0.215 to −0.116)** | **−0.084 (−0.119 to −0.049)** | **−0.084 (−0.116 to −0.052)** |
| Female vs male | **−0.039 (−0.061 to −0.018)** | **−0.033 (−0.048 to −0.019)** | **−0.030 (−0.043 to −0.017)** |
| White vs non-white | 0.003 (−0.035 to 0.041) | 0.005 (−0.019 to 0.030) | 0.005 (−0.017 to 0.028) |
| Single vs married or cohabiting | **−0.056 (−0.089 to −0.023)** | **−0.026 (−0.048 to −0.004)** | **−0.021 (−0.041 to −0.001)** |
| Separated, divorced or widowed vs married or cohabiting | **−0.092 (−0.118 to −0.067)** | **−0.052 (−0.073 to −0.030)** | **−0.047 (−0.066 to −0.028)** |
| Degree or higher vs below degree | **0.066 (0.041 to 0.091)** | 0.013 (−0.005 to 0.030) | 0.011 (−0.005 to 0.027) |
| IMD Q2 vs Q1 | −0.030 (−0.061 to −0.000) | −0.014 (−0.036 to 0.009) | −0.011 (−0.032 to 0.009) |
| IMD Q3 vs Q1 | **−0.042 (−0.076 to −0.009)** | −0.021 (−0.044 to 0.001) | −0.019 (−0.040 to 0.002) |
| IMD Q4 vs Q1 | **−0.064 (−0.095 to −0.033)** | **−0.064 (−0.086 to −0.041)** | **−0.058 (−0.078 to −0.037)** |
| IMD Q5 vs Q1 | **−0.093 (−0.126 to −0.061)** | **−0.065 (−0.088 to −0.042)** | **−0.055 (−0.076 to −0.035)** |
| Limiting longstanding illness vs none | **−0.490 (−0.513 to −0.466)** | **−0.364 (−0.386 to −0.343)** | **−0.338 (−0.357 to −0.320)** |
| Constant | **1.344 (1.282 to 1.406)** | **1.176 (1.137 to 1.215)** | **1.141 (1.104 to 1.177)** |

Coefficients whose 95% confidence interval excludes zero are shown in bold. This is a reading aid only and is not a claim about the importance of an association; the interval itself, not the bolding, carries the inferential content

Same covariates and complete-case sample as Table S6, with censoring at an upper limit of 1

No Tobit model is fitted for the mapped column. It is a conditional expectation with no mass at 1, its maximum being 0.9885, so there is no ceiling to censor and a censored model would be misspecified

Tobit coefficients are on the latent scale and are larger in magnitude than the corresponding linear regression coefficients in Table S6; they are not directly comparable with them

Value sets: UK EQ-5D-3L, Dolan 1997; UK EQ-5D-5L, Rowen et al. 2026; England EQ-5D-5L, Devlin et al. 2018

IMD Index of Multiple Deprivation

**Table S8** Sensitivity analysis: survey-weighted generalised linear model for the EQ VAS, Health Survey for England 2017

| **Covariate** | **Generalised linear model** | **Linear regression** |
| --- | --- | --- |
| Age band 2 vs band 1 | −0.0114 (−0.1506 to 0.1279) | −0.2195 (−2.3160 to 1.8770) |
| Age band 3 vs band 1 | −0.0440 (−0.1795 to 0.0916) | −0.6912 (−2.7575 to 1.3751) |
| Age band 4 vs band 1 | −0.0861 (−0.2218 to 0.0495) | −1.3745 (−3.4591 to 0.7101) |
| Age band 5 vs band 1 | −0.0388 (−0.1792 to 0.1017) | −0.6173 (−2.7788 to 1.5442) |
| Age band 6 vs band 1 | 0.0128 (−0.1286 to 0.1542) | 0.3282 (−1.8480 to 2.5045) |
| Age band 7 vs band 1 | −0.1640 (−0.3124 to −0.0156) | −2.8116 (−5.2049 to −0.4183) |
| Female vs male | −0.0510 (−0.1041 to 0.0021) | −0.7933 (−1.6192 to 0.0326) |
| White vs non-white | 0.1029 (0.0020 to 0.2039) | 1.5564 (−0.0651 to 3.1779) |
| Single vs married or cohabiting | −0.0857 (−0.1685 to −0.0030) | −1.3319 (−2.6769 to 0.0131) |
| Separated, divorced or widowed vs married or cohabiting | −0.1722 (−0.2529 to −0.0915) | −3.0577 (−4.4859 to −1.6295) |
| Degree or higher vs below degree | 0.0475 (−0.0145 to 0.1095) | 0.6585 (−0.2702 to 1.5871) |
| IMD Q2 vs Q1 | −0.0680 (−0.1554 to 0.0194) | −1.0046 (−2.2789 to 0.2698) |
| IMD Q3 vs Q1 | −0.0828 (−0.1747 to 0.0090) | −1.2164 (−2.5747 to 0.1419) |
| IMD Q4 vs Q1 | −0.1638 (−0.2481 to −0.0794) | −2.4944 (−3.7952 to −1.1936) |
| IMD Q5 vs Q1 | −0.2724 (−0.3592 to −0.1855) | −4.3958 (−5.7938 to −2.9977) |
| Limiting longstanding illness vs none | −1.0694 (−1.1306 to −1.0081) | −19.8233 (−21.0626 to −18.5840) |
| Constant | 1.7861 (1.6086 to 1.9636) | 85.9804 (83.2387 to 88.7221) |

The generalised linear model uses a binomial family scaled to 100 with a logit link. This fractional-response specification respects the bounded 0 to 100 range of the EQ VAS

Coefficients are on the link scale and are not directly comparable with the linear regression column, which is reproduced from Table S6 for reference only

Covariates and complete-case sample are as in Table S6

IMD Index of Multiple Deprivation

**Table S9** Contribution of each EQ-5D dimension to the difference between the UK and England EQ-5D-5L value sets, Health Survey for England 2017

| **Dimension** | **All respondents** | **No dimension rated level 4 or worse** | **Any dimension rated level 4 or worse** |
| --- | --- | --- | --- |
| **Panel A. Contribution by dimension** | | | |
| Dimension | All respondents | No dimension at level 4 or worse | Any dimension at level 4 or worse |
| Mobility | 0.0049 (0.0046 to 0.0051) | 0.0036 (0.0033 to 0.0038) | 0.0180 (0.0169 to 0.0191) |
| Self-care | 0.0010 (0.0009 to 0.0011) | 0.0004 (0.0003 to 0.0004) | 0.0068 (0.0062 to 0.0075) |
| Usual activities | −0.0020 (−0.0022 to −0.0019) | −0.0006 (−0.0007 to −0.0005) | −0.0161 (−0.0170 to −0.0152) |
| Pain or discomfort | −0.0015 (−0.0021 to −0.0009) | 0.0038 (0.0036 to 0.0039) | −0.0539 (−0.0585 to −0.0492) |
| Anxiety or depression | 0.0035 (0.0030 to 0.0040) | 0.0054 (0.0049 to 0.0059) | −0.0148 (−0.0174 to −0.0123) |
| Total difference (sum of the five contributions) | 0.0058 (0.0049 to 0.0067) | 0.0125 (0.0119 to 0.0131) | −0.0599 (−0.0653 to −0.0546) |
| n | 7,169 | 6,439 | 730 |
| **Panel B. Total difference by number of dimensions at level 4 or worse** | | | |
| Severe dimensions | n | Difference (95% CI) |  |
| 0 | 6,439 | 0.0125 (0.0119 to 0.0131) |  |
| 1 | 322 | −0.0302 (−0.0365 to −0.0238) |  |
| 2 | 182 | −0.0569 (−0.0674 to −0.0463) |  |
| 3 | 132 | −0.0892 (−0.1005 to −0.0780) |  |
| 4 | 70 | −0.1295 (−0.1432 to −0.1158) |  |
| 5 | 24 | ‡ |  |

Cells show the survey-weighted mean contribution and its 95% confidence interval, to four decimal places. A positive contribution indicates that the dimension raises the UK-scored index above the England-scored one

Because each index is one minus the sum of five dimension decrements, the difference between them equals the sum of the five dimension-level differences exactly. The decomposition therefore has no residual term and the five contributions sum to the total before rounding

Each additional dimension rated level 4 or worse changes the difference by −0.036 (p < 0.001)

The comparison is within respondent, so cohort and descriptive system are held constant by construction and the whole difference is attributable to the value set: the elicited population, the elicitation method and the model

Value sets: UK EQ-5D-5L, Rowen et al. 2026; England EQ-5D-5L, Devlin et al. 2018.

‡ Suppressed because the cell contains fewer than 25 unweighted observations

CI confidence interval

**Table S10** Contribution of sample composition to the difference between Health Survey for England 2014 and 2017

| **Model** | **Wave coefficient (95% CI)** | **Share of unadjusted difference explained, %** |
| --- | --- | --- |
| **Panel A. Wave coefficient as adjustment is added, outcome on the UK EQ-5D-3L scale** | | |
| Model | Wave coefficient (95% CI) | Share of the unadjusted difference explained, % |
| Unadjusted | −0.0288 (−0.0376 to −0.0200) | 0.0 |
| Adjusted for age band and sex | −0.0266 (−0.0350 to −0.0182) | 7.7 |
| Adjusted for age, sex, ethnicity, marital status, education and IMD quintile | −0.0283 (−0.0360 to −0.0206) | 1.8 |
| **Panel B. HSE 2014 standardised to the HSE 2017 age and sex structure** | | |
| Quantity | UK EQ-5D-3L scale | EQ VAS |
| HSE 2014, crude | 0.867 | 78.5 |
| HSE 2014, standardised to HSE 2017 | 0.864 | 78.2 |
| HSE 2017, crude | 0.838 | 79.2 |
| Crude difference (2017 minus 2014) | −0.0288 | 0.7 |
| Standardised difference | −0.0264 | 1.0 |
| Attributable to age and sex composition | −0.0024 | −0.3 |

Both waves are placed on the UK EQ-5D-3L scale: HSE 2014 is scored directly and HSE 2017 is converted using the crosswalk. Without a common scale the wave difference cannot be decomposed

The crosswalk rather than the mapping is used as the common-scale outcome because it is deterministic. The mapping is conditional on age and sex, so a wave comparison of age-conditional predictions adjusted for age and sex would be partly circular

The EQ VAS column is the only outcome recorded identically in both waves and independent of any value set, and serves here as a value-set-independent anchor

Sample composition can be removed, but the descriptive system and the period cannot be separated, because no respondent completed both instruments. The residual after adjustment combines period change with instrument effect and this analysis cannot apportion it between them

Value sets: UK EQ-5D-3L, Dolan 1997

HSE Health Survey for England, CI confidence interval

**Figure S1** Distribution of EQ-5D index scores under each scoring route, Health Survey for England 2014 and 2017

 Panel A: Health Survey for England 2014, UK EQ-5D-3L value set. Panels B to E: Health Survey for England 2017, scored with the UK EQ-5D-5L value set, the England EQ-5D-5L value set, the reference-case mapping and the crosswalk

All panels use identical bins of width 0.025 and a common vertical scale. The dashed line marks an index value of 1.000

Panel D does not reach 1.000 because the mapping returns a conditional expectation whose maximum is 0.9885; it therefore has no ceiling

Value sets: UK EQ-5D-3L, Dolan 1997; UK EQ-5D-5L, Rowen et al. 2026; England EQ-5D-5L, Devlin et al. 2018

**Figure S2** Survey-weighted mean EQ-5D index scores by Index of Multiple Deprivation quintile, Health Survey for England 2014 and 2017

 Panel A: series on the UK EQ-5D-3L scale, comprising Health Survey for England 2014 scored directly and Health Survey for England 2017 converted using the crosswalk. Panel B: the two EQ-5D-5L series in Health Survey for England 2017

The panels use different value sets, so absolute index levels are not directly comparable between panels. Comparisons should focus on deprivation gradients within each panel

Error bars are 95% confidence intervals from survey-weighted estimates. IMD quintiles are derived from IMD 2010 for 2014 and from IMD 2015 for 2017

IMD Index of Multiple Deprivation
